# Effects of varying levels of coated cysteamine hydrochloride in diet on growth performance and carcass quality of steers

**DOI:** 10.5713/ab.24.0863

**Published:** 2025-04-28

**Authors:** Watcharawit Meenongyai, Kannika Wongpanit, Pichad Khejornsart, Piyamas Phongkaew, Unchan Traithilen, Naroon Waramit, MD. Maksudul Haque Helali, Alimul Islam Shimul, Abdullah Al Mamun

**Affiliations:** 1 Faculty of Natural Resources and Agro-Industry, Kasetsart University Chalermphrakiat Sakon Nakhon Province Campus, Sakon Nakhon, Thailand; 2 School of Integrated Science, Kasetsart University, Bangkok, Thailand; 3 Department of Agronomy, Faculty of Agriculture at Kamphaeng Saen Campus, Kasetsart University, Nakhon Pathom, Thailand; 4 Faculty of Animal Husbandry, Bangladesh Agricultural University, Mymensingh, Bangladesh

**Keywords:** Carcass Quality, Cysteamine, Fatty Acids, Growth Performance, Muscle Fiber

## Abstract

**Objective:**

This study aimed to evaluate the effects of different levels of coated cysteamine HCl (CSH) supplementation on growth performance, carcass traits, and meat quality in Charolais crossbred cattle.

**Methods:**

Twenty-four Charolais crossbred steers, aged 24–30 months with an initial body weight of 418±31 kg, were assigned to three dietary treatments: 0% (control), 0.5%, or 1.0% CSH in concentrate. The steers were fed the experimental diets for 200 days.

**Results:**

Increased CSH levels in concentrate diets led to significant increases (p<0.05) in body weight, hot carcass weight, cold carcass weight, and dressing percentage. CSH supplementation decreased shear force values and cooking loss (p<0.05). Meat lightness was significantly greater in the 1.0% CSH-supplemented group (p<0.05). With increasing CSH levels, the proportion of high-value cuts, such as rib set and T-bone, increased (p<0.05), while the proportion of lower-value cuts, including macreuse, shank, and tendons, decreased (p<0.05). Non-carcass weights, including the feet, head, digestive tract, and scraps, were significantly higher in the 1.0% CSH-supplemented diet (p<0.05). The meat’s chemical composition did not differ significantly among the treatment groups (p>0.05). Muscle fiber diameter was significantly larger in the 1.0% CSH-supplemented group (p<0.05). Monounsaturated fatty acids increased (p<0.05) with higher CSH levels, whereas polyunsaturated and saturated fatty acids exhibited a significant decrease (p<0.05).

**Conclusion:**

Dietary supplementation with CSH enhances growth rate, carcass traits, and meat quality in steers. An inclusion level of 1.0% CSH in concentrate is the optimum feeding dosage, demonstrating its potential as an effective feed additive for enhancing beef production efficiency.

## INTRODUCTION

Beef is a superior protein source, supplying all essential amino acids and crucial micronutrients [1]. By 2033, global beef consumption is projected to increase by 11%, with production expected to reach 81 million tons (carcass-weight equivalent) by that year. This growth will be driven by factors such as higher carcass weights, improved genetics, enhanced farm management practices, and significant growth in Asian countries such as China and India, along with expanded production capacity in Australia [2]. As of 2023, Thailand’s total beef cattle population was 9.65 million head, with only 3.29% comprising crossbred cattle raised in intensive systems for the premium market, selected based on marbling scores [3]. Crossbred Charolais×Brahman×Thai native cattle (*Bos taurus*×*Bos indicus*) are particularly favored in these premium markets for their superior meat quality and adaptability, combining the strengths of both breed types to ensure productivity across diverse farming conditions [4]. As demand for beef grows, farmers and producers seek innovative methods to boost cattle production and efficiency. Growth-promoting technologies, such as antibiotics, beta-agonists, and hormone implants, boost meat production efficiency. However, these technologies raise concerns over residue-related risks, including allergies, antimicrobial resistance, and carcinogenic effects [5], and potential carcinogenic effects, thus presenting a critical challenge for producers in balancing productivity with consumer safety.

Ongoing research is focusing on the use of feed additives to improve animal growth performance, with cysteamine emerging as a notable candidate. Cysteamine is a bioactive amino molecule, a decarboxylation product of cysteine, featuring both an amine and a thiol group in its structure, with the chemical formula C_2_H_7_NS [4]. It has demonstrated effectiveness in promoting growth and enhancing efficiency across various livestock species [6]. Its mode of action involves reducing the plasma concentration of somatostatin (SS) by interacting with the disulfide bonds of the SS hormone, thereby disrupting its function and leading to increased somatotropin (growth hormone) activity [7]. However, cysteamine hydrochloride, commonly used in animal diets for its stability and solubility, faces challenges such as bitterness, odor, moisture absorption, and instability [4]. Encapsulation technologies, such as coated cysteamine HCl (CSH), address these issues by enabling controlled release, enhancing pharmacokinetics, safety, and overall efficacy [6].

A study on beef cattle by Meenongyai et al [8] demonstrated that cysteamine supplementation effectively enhanced feed intake and improved the nutritional status of growing heifers. Similarly, research by Sun et al [9] found that steers fed CSH at 20 g/head/day or 80 mg/kg body weight (BW) exhibited a 16% increase in body weight gain (BWG). However, data on the effects of CSH on meat quality and carcass traits in beef cattle are currently limited. Therefore, this study aimed to investigate the impact of different levels of CSH supplementation on growth performance, carcass traits, and meat quality in steers. We hypothesize that CSH inclusion will improve growth rates, carcass traits, and meat quality in steers. Identifying optimal supplementation levels could refine feeding strategies, promoting growth and enhancing carcass quality.

## MATERIALS AND METHODS

### Animals, dietary treatments, and data collection

A total of 24 Charolais crossbred steers (50% Charolais, 25% Brahman, and 25% Thai native), aged 24 to 30 months with an average BW of 418±31 kg, were assigned to a randomized complete block design. The steers were stratified into eight blocks based on their age, with each block consisting of three animals. Within each block, the animals were randomly allocated to one of three dietary treatments. At the beginning of the trial, the steers were dewormed with anthelmintic injections following the manufacturer’s instructions (Ivomec-F, Vet Inter Pharma, Samut Prakan, Thailand). Each steer was housed in an individual pen measuring 3 m×4 m, with a concrete floor, feed trough, and an automated water dispenser. The steers underwent a 14-day adaptation period to their experimental diet before the commencement of the 200-day experimental period. To assess BWG, weights were recorded on day 0 of the adaptation phase and then monthly thereafter.

To fulfill the nutritional needs of steers, the experimental diet was formulated based on the guidelines set by NASEM [10]. Three levels of CSH supplementation were included in the dietary treatments: 0%, 0.5%, and 1.0%, equating to 0, 75, and 150 mg/kg BW, respectively. The CSH, containing 27% active ingredient, was provided by King Techina (Zhejiang, China), AMCOVET (Bangkok, Thailand). The concentrate, which consisted of 12.0% crude protein (CP), was offered at 1.5% of BW, along with fermented cassava pulp at 1.0% of daily BW. Additionally, rice straw was made available ad libitum. The diet was provided in two equal portions, at 8:00 a.m. and 5:00 p.m. To determine dry matter intake (DMI), refusal weights were measured and recorded daily before the morning feeding. The feed ingredients and their chemical composition are detailed in Table 1.

### Carcass measurement

Following a 200-day feeding trial, all steers were slaughtered after a 24-hour fasting period at Nakhon Phanom Beef (Nakhon Phanom, Thailand). At the time of slaughter, hot carcass weight (HCW) was recorded, and the weights of offal items were measured to assess the impact of the diet on by-product yields. The carcasses were chilled at 1±1°C for seven days, after which the cold carcass weight (CCW) was recorded, and chilling loss was determined. Fabrications were performed at the 12th–13th rib to measure ribeye area, fat thickness, and marbling score. Following the collection of carcass data, all carcasses were fabricated according to French standards [11], and subprimal yields were determined as a percentage of the chilled carcass weight. A 4-rib section (ribs 9–12) was fabricated from the primal rib cut, vacuum-packaged per plant specifications, and transported to Kasetsart University Chalermprakiat Sakon Nakhon Province Campus (76 km away) while maintaining a cold chain. On the same day (day −7 postmortem), the 10th and 11th ribs were separated from the previously collected 9–12 rib sections for objective color measurement. Objective meat color (CIE -L*-a*-b*) analysis was performed using six replicates taken from the uncovered longissimus thoracis (LT; 10th rib) after approximately 30 min of blooming. Color measurements were conducted using a Minolta CR-300 (Minolta, Osaka, Japan). The LT was then deboned and cut into steaks (2.54 cm thickness) from posterior to anterior for further evaluations, including Warner-Bratzler shear force, cooking loss, muscle fiber characteristics (fiber diameter and length), proximate analysis, and fatty acid profile. The steaks were vacuum-sealed and individually stored at −20°C for subsequent analyses.

### Warner-Bratzler shear force determination

Following the method outlined by Gök et al [12], the steaks were thawed at 4°C for 24 hours before being cooked sous vide for 75 min at 80°C in a preheated water bath. The beef samples were cut into uniform pieces, weighed (initial weight), and then boiled. After cooking, the samples were cooled under running tap water for 30 min to reach room temperature (25°C). The steaks were then removed from the vacuum bags, drained, and weighed to calculate cooking loss. The samples were trimmed and sectioned into 1.27 cm diameter cores. Shear force measurements were carried out using a TA-XT2i texture analyzer equipped with a Warner-Bratzler blade and a 500 N load cell, set to a crosshead speed of 200 mm/min [13]. The peak shear force was recorded for each core, and the average value was calculated for each sample.

### Proximate analysis of meat sample

The meat samples were thawed at 0°C for 24 h, during which visible external fat and connective tissue were removed. The separable lean was then hand-chopped and thoroughly blended using a meat blender (HR2223/00 Series-5000, Philips, Shanghai, China). The chemical composition of the meat samples was determined in triplicate following AOAC [14] methods. Moisture content was measured by drying the samples in desiccated aluminum pans at 55°C for 72 hours (Memmert, Schwabach, Germany). CP was quantified using the Kjeldahl method. After moisture determination, the dried samples were analyzed for total fat content using a Soxhlet extractor (Soxtherm, C. Gerhardt, Königswinter, Germany). Ash content was measured by burning the sample in a muffle furnace at 550°C for 12 hours (MF 110 NUVE, Nüve, Ankara, Turkey).

### Determining muscle fiber diameter and sarcomere length

After thawing the samples for 24 hours at 0°C, two 5 mm×5 mm×5 mm cores were taken out of the middle of each sample, one for longitudinal analysis and one for transverse examination. The cores were fixed in 2.5% glutaraldehyde in phosphate buffer at 4°C for 24 hours and then post-fixed in 1% osmium tetroxide for 2 hours at room temperature [15]. After rinsing and dehydrating through graded ethanol concentrations, the samples were cryopreserved in liquid nitrogen. One core was sliced transversely and the other longitudinally. A 1-mm-thick slice from each core was dried using liquid CO_2_ in a critical point dryer (HCP-2, Hitachi, Tokyo, Japan) [16]. The dried samples were gold-coated and examined with a scanning electron microscope (JSM-6020LV, JEOL, Tokyo, Japan) at 5 kV [17]. Micrographs were taken at 500× magnification for transverse sections and 5,000× for longitudinal sections. Using ImageJ software, muscle fiber diameters and sarcomere lengths were measured from five randomly selected images per slice. Ten fibers and sarcomeres per image were assessed for diameter and length, respectively.

### Fatty acid analysis

Lipid extraction was performed following the method of Folch et al [18]. To 15 g of the sample, 90 mL of a 2:1 chloroform-methanol mixture and 25 μg of butylated hydroxyanisole in 98% ethanol were added. The mixture was homogenized for 120 seconds using a Nissei AM-8 homogenizer. After filtration through Whatman No. 1 paper, the extract was transferred to a separating funnel, followed by the addition of 5 mL 0.58% NaCl and 30 mL deionized water. The layers were separated, and the organic phase was dried with anhydrous Na_2_SO_4_. Lipids were concentrated using a rotary evaporator at 40°C and stored at −20°C in an amber vial purged with nitrogen until methylation. The methylation process, based on Ostrowska et al [19], involved the addition of 25 mg of lipid to a screw-cap tube with 1.5 mL of 0.5 N NaOH in methanol and 15 mL of methanol. The mixture was flushed with nitrogen, sealed, and incubated at 100°C for 5 min. After cooling, 2 mL of 14% BF_3_/MeOH was added under nitrogen and incubated again at 100°C for 5 min. After cooling, 10 mL of deionized water was added, and the mixture was centrifuged at 2,800×g for 15 min at 10°C. The hexane layer containing the methylated fatty acids was collected for gas chromatography analysis.

Fatty acid methyl esters were analyzed using a Hewlett Packard GC system (HP 7890A) with an auto-sampler and a DB-wax capillary column (0.25 mm ID, 60 m length, 0.25 μm film thickness). There was a 100:1 split ratio and a 1 μL injection volume. The column temperature was programmed as follows: 70°C for 4 min, ramped to 175°C at 13°C/min with a 27-minute hold, and then to 240°C at 3°C/min with a 30-minute hold. The injector was set at 240°C, with helium as the carrier gas at 1.0 mL/min. Fatty acids were identified using a mass spectrometer (Model 7000B) in electron ionization mode at 70 eV and ion source temperature of 230°C. Mass spectrometry detection covered 35–550 m/z. Identification was based on the Wiley library (NIST MS Search, version 2.0g), and fatty acid levels were quantified by peak area and expressed as percentages of the total fatty acid content.

### Statistical analysis

All data were analyzed using the PROC MIXED procedure in SAS (version 9.0; SAS Institute, Cary, NC, USA). The statistical model included dietary treatment as a fixed effect, while block (n = 8) was treated as a random effect. Initial body weight (BWI) was initially included as a covariate but was removed from the model if found non-significant (p>0.05). The general model used was:


(1) $$Y_{ij}=\mu +T_{i}+B_{j}+r(Xij-X)+e_{ij}$$

Where *Y_ij_* = observed response variable, μ = overall mean, *T_i_* = fixed effect of dietary treatment (*i* = 0.0%, 0.5%, and 1.0% CSH), *B_j_* = random effect of block (*j =* 1,2,...,8), *r* = regression coefficient of *Y_ij_* on the covariate *X_ij_*, *X_ij_* = covariate (BWI), *X̄* = overall mean of *X_ij_*, *e_ij_* = residual error.

For growth performance data (BW and average daily gain [ADG]), which involved repeated measurements, a repeated measures approach was applied using an autoregressive covariance structure [AR(1)] to account for within-subject correlations. The statistical model was:


(2) $$Y_{ijk}=\mu +T_{i}+B_{j}+A_{jk}+{\left(T\times Time\right)}_{ik}+Time_{k}+\rho (Xij-X)+ɛijk$$

Where *A_jk_* = random effect of animal (*k*) nested within block, (*T×Time*)*_ik_* = interaction between treatment and time, *Time_k_* = repeated measure effect of time, and other terms are as defined previously. Least square means (LSMeans) were compared using the PDIFF option in the LSMeans statement. Multiple comparisons were performed using Tukey’s HSD test to control for Type I error, with differences considered statistically significant at p<0.05. Trends were evident in the p-values between 0.05 and 0.10. Orthogonal polynomial contrasts were used to evaluate linear and quadratic trends of CSH levels. Data normality was assessed using the PROC UNIVARIATE procedure. To assess the normality of residuals, Shapiro-Wilk and Kolmogorov-Smirnov tests were conducted. If data violated normality assumptions (p<0.05), they were transformed using logarithmic (log10) or square root transformation before reanalysis. Homogeneity of variance was verified using Levene’s test, and outliers were checked using studentized residuals (>±3 SD). If transformations did not normalize the data, nonparametric methods were considered.

## RESULTS AND DISCUSSION

### Dry matter intake and growth performance

The inclusion of varying levels of CSH supplementation in the concentrate did not have a significant effect on DMI or DMI as a percentage of BW in steers (p>0.05), as outlined in Table 2. These results are in agreement with those obtained by Meenongyai et al [4] and Sun et al [9], who also observed no significant changes in DMI with CSH supplementation in cattle diets. However, previous studies have reported both positive [20] and negative [9] effects of CSH supplementation on DMI in livestock. The feed conversion ratio (FCR) was not significantly affected by coated CSH supplementation (p> 0.05). However, a significant linear reduction in FCR was observed (p = 0.04). Although the difference was not statistically significant (p>0.05), FCR numerically improved by 10.3% and 21.3% in the 0.5% and 1.0% CSH groups, respectively, compared to the control. These results align with the findings of Sun et al [9], who reported a non-significant reduction of 15.24% in FCR in steers supplemented with 80 mg/kg BW of CSH over a 56-day period. These findings are consistent with those of Sun et al [9], who reported a non-significant 15.24% reduction in FCR in steers supplemented with 80 mg/kg BW of CSH over a 56-day period. Although total feed costs increased with higher levels of CSH in the diet (p<0.05), there were no significant differences in feed cost per kilogram of gain among the treatments (p>0.05), likely due to the increased BWGs observed in steers fed CSH-supplemented diets.

The BW of the steers at 60, 120, and 150 days showed a significant increase with 1% CSH supplementation (p<0.05), as presented in Table 2. Additionally, significant linear increases were observed at 60, 90, 120, 150, and 200 days (p<0.05). In addition, the ADG during the 0–60, 0–120, and 0–150-day periods showed significant increases with 1% CSH supplementation (p<0.05). Furthermore, significant linear increases were observed across the 0–60, 0–90, 0–120, 0–150, and 0–200-day periods (p<0.05). These findings suggest that the effect of CSH supplementation on growth involves the suppression of SS, leading to an increase in circulating growth hormone levels, thereby enhancing growth [6,7]. Long-term studies on CSH feeding are currently unavailable. However, a previous study reported that dietary supplementation of 30% CSH at 20 g/day during a short-term fattening period of 63 days resulted in a 15.68% increase in BWG in beef cattle [20]. Similarly, Sun et al [9] reported a 16.03% improvement in BWG in steers supplemented with CSH at a dosage of 80 mg/kg BW over a 56-day period. In the present experiment, the cattle were raised for an extended duration of 200 days. It was observed that growth increased significantly during the initial 150 days. However, as the cattle approached maturity, growth stabilized, leading to a uniform growth rate during the final stage of the feeding period.

### Carcass characteristics

The carcass characteristics of steers fed diets supplemented with different levels of CSH are presented in Table 3. HCW and CCW were significantly increased with 1.0% CSH supplementation (p<0.05). Furthermore, the dressing percentage (DP) in the 1.0% CSH group was significantly higher than in the 0.5% CSH group (p<0.05). Linear increases in HCW and CCW were observed with higher levels of CSH supplementation (p<0.05), while a quadratic effect was observed for DP (p<0.05). However, no significant differences were found in rib-eye area across the treatment groups (p>0.05). Previous studies have demonstrated that cysteamine can enhance carcass performance and meat quality in pigs [21]. Wu et al [7] also found that finishing lambs receiving diets supplemented with CSH at 20–60 mg/kg BW showed a linear increase in both HCW (1.9%–4.5%) and net meat yield (4.3%–10.6%). However, the effects of dietary CSH concentrations may vary depending on the species and diet type. For cattle, 80 mg CSH/kg BW did not significantly affect BWG [9]. The present study confirms improved carcass weight in beef cattle, reflecting enhanced muscle growth with increasing CSH levels.

Research on pigs has shown that dietary CSH supplementation above 27 mg/kg BW can reduce backfat thickness and increase lean tissue proportions [22]. In the present study, no significant differences were found in backfat thickness or marbling scores between the treatment groups (p>0.05), suggesting that CSH supplementation did not affect carcass fat content in long-term finishing steers. Furthermore, the shear force value was significantly reduced with 1.0% CSH supplementation (p<0.05). A linear decrease in shear force value was also observed with increasing dietary CSH concentrations (p<0.05), suggesting an enhancement in meat tenderness. Muscle fiber diameter (Table 4) was greater in the 1.0% CSH group and increased linearly with higher CSH supplementation levels (p<0.05). The reduction in shear force observed in the 1.0% CSH-supplemented group suggests that the increased fat content, as indicated by the numerically higher meat fat composition (Table 4), likely contributed to the observed improvement in meat tenderness. Additionally, CSH may reduce excessive protein crosslinking, further enhancing tenderness. According to Jeitner [23], cysteamine inhibits the formation of crosslinked proteins, facilitating the digestion of previously crosslinked proteins.

In this study, cooking loss was significantly lower in the 1.0% CSH group compared to the control group (p<0.05) and exhibited a linear decrease with increasing dietary CSH concentrations (p<0.05). Despite a reduction in moisture content, the linear decrease in cooking loss (p<0.05) indicates that CSH supplementation improves the meat’s capacity to retain water during cooking. According to Pang et al [24], the main reasons for water loss during cooking are temperature-induced denaturation and structural alterations in proteins. Therefore, CSH may reduce water loss by stabilizing muscle protein structures through interactions with sulfhydryl groups [25], thereby minimizing denaturation. This process helps retain water, enhancing meat juiciness and quality. However, there were no evident differences in carcass chilling loss across the treatment groups (p>0.05). Our findings align with a previous study, which reported that incorporating CSH into diets had no impact on drip loss in finishing lambs [7].

The lightness (L*) of the longissimus muscle was significantly higher in the 1.0% CSH group (p<0.05) and exhibited a linear increase with higher dietary CSH concentrations (p<0.05). This improvement is likely attributed to the antioxidant properties of CSH, which may have mitigated oxidative damage [26]. The redness (a*) value increased quadratically with rising dietary CSH levels (p<0.05). However, CSH may reduce redness by promoting myoglobin oxidation and denaturation, converting oxymyoglobin and deoxymyoglobin into metmyoglobin, as noted by Bak et al [27]. Yellowness (b*) was not significantly affected by CSH supplementation (p> 0.05). These findings align with those of Bai et al [28], who concluded that dietary supplementation with CSH can improve antioxidant status and delay meat browning by increasing glutathione levels and antioxidant activity during extended cold storage.

### Subprimal cut

The effects of varying levels of CSH supplementation on the subprimal cuts of steers are detailed in Table 5. The proportion of rib set, stew meat, and T-bone cuts was significantly higher in the 1.0% CSH group compared to the control group (p<0.05) and exhibited a linear increase with higher dietary CSH concentrations (p<0.05). In contrast, the 1.0% CSH group showed lower proportions of macreuse (clod), paleron (flat iron), fore shank, silver shank, knuckle, bottom round, hide shank, nerveux (blade), and tendon (p<0.05). Additionally, the proportion of scraps decreased with increasing CSH levels (p<0.05). However, no significant differences were observed in the proportions of chuck, brisket, short rib, hampe, sirloin, tri-tip, bavette aloyau (flap), bavette flanchet (flank), bone, or fat among the treatment groups (p>0.05). In finishing pigs, CSH supplementation has shown favorable effects on carcass traits and meat attributes [22]. While improvements in daily weight gain have been reported in beef cattle (15%), sheep (28%), yaks (54%), steers (16%), and lambs (28%), limited information exists regarding its influence on carcass characteristics in ruminants [6]. This study indicates that CSH supplementation significantly impacts subprimal cut distribution in steers, enhancing the proportions of high-value cuts such as rib set, T-bone, and stew meat while reducing lower-value cuts such as macreuse, tendons, and scraps. These shifts are likely attributed to improved muscle development, consistent with the findings of Bai et al [29], who reported that CSH enhances protein deposition in finishing pigs by upregulating amino acid transporter expression. These findings reinforce the potential of CSH to enhance carcass quality and optimize meat cut distribution.

### Non-carcass weight

The effects of different CSH levels in diets on non-carcass weights of steers are summarized in Table 6. The weight of the feet, head, and scraps was significantly higher in the 1.0% CSH-supplemented diet (p<0.05), which may be attributed to the effect of CSH on growth hormones, thereby promoting tissue and organ development [7]. This observation is consistent with the findings of Cattelam et al [30], who reported that diet contributes to variations in the composition of the feet and head. Vaz et al [31] further highlighted the economic significance of the feet in the slaughter industry, particularly for their tendons, which are exported to Asian markets. The head, which makes up around 3.57% of the cattle’s empty BW, is usually deboned and used to make sausages, minced meat, and other processed foods [30]. Moreover, the digestive tract weight was significantly higher in the 0.5% CSH-supplemented diet (p<0.05) and increased linearly with higher CSH concentrations in the diet (p<0.05). The enhanced development of the gastrointestinal tract in steers fed CSH is likely due to the higher DMI, which may have slowed the rate of passage and facilitated greater development of this compartment [32]. The liver weight tended to increase with 0.5% CSH supplementation in the diet (p = 0.06), and exhibited a linear trend with higher CSH concentrations in the diet (p = 0.08). Cook et al [33] indicated that the liver absorbs about 80% of propionate for conversion into glucose and is involved in ammonia uptake and conversion to urea, so the higher (though nonsignificant) DMI in cattle fed CSH may provide more energy, potentially contributing to increased liver development. However, no significant differences were observed for the skin, tail, or bile weights (p>0.05), possibly due to its relation to the function of this component.

### Meat composition

The chemical composition of meat from steers fed diets supplemented with different amounts of CSH is presented in Table 4. The moisture content decreased linearly with increasing CSH levels in the diet (p<0.05), while CP content increased quadratically (p<0.05). The reduction in moisture content might have resulted from increased muscle fiber diameter, which decreases inter-fiber space and lowers water-holding capacity (WHC). Şirin et al [34] found a negative correlation between type I muscle fiber diameter and WHC in the longissimus dorsi (LD) muscle (r = −0.411), suggesting that larger muscle fibers contribute to lower WHC. However, there was no significant difference in the amount of fat or ash across the treatment groups (p>0.05). In addition, dietary supplementation with CSH has been shown to enhance lipolysis and suppress lipogenesis, thereby reducing fat deposition in the carcass [22]. Despite these effects, CSH supplementation did not significantly alter the meat’s chemical composition in the current study. These results are consistent with those of Tao et al [22], who reported no significant effect on fat percentage in the carcasses of pigs supplemented with CSH during the finishing period. These observed differences are attributable to the variations in the animal type, dose, or duration of supplements.

### Muscle fiber characteristics

**Figure 1:**
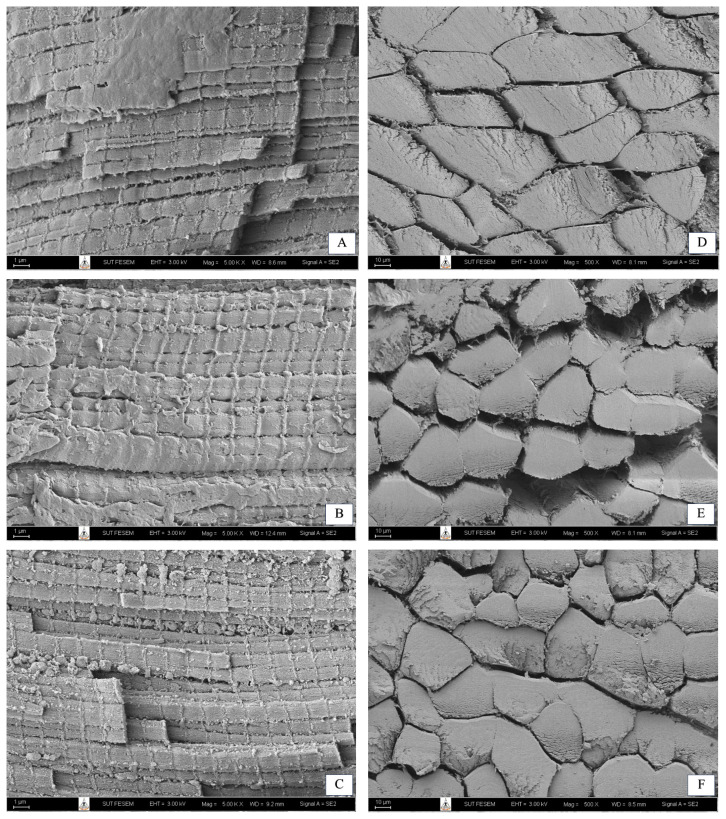
SEM micrographs of raw LT muscle stored for 7 days at 1±1°C and frozen at −20°C: longitudinal sections (×5,000). (A) Control, (B) 0.5% CSH, (C) 1.0% CSH; transverse sections (×500): (D) Control, (E) 0.5% CSH, (F) 1.0% CSH. SEM, scanning electron microscopy; LT, longissimus thoracis; CSH, coated cysteamine hydrochloride.

The muscle fiber characteristics of raw beef from steers fed diets with varying levels of CSH are presented in Table 4. Myofibril microstructures of raw LT muscle are depicted in transverse and longitudinal section micrographs (Figure 1). The sarcomere length showed a quadratic trend in response to increasing CSH concentrations in the diet (p = 0.05), suggesting that CSH supplementation enhances meat tenderness. Longer sarcomeres are positively correlated with improved tenderness, as they facilitate better muscle relaxation during cooking, resulting in a softer texture [35]. Additionally, muscle fiber diameter was significantly greater in the 1.0% CSH-supplemented group (p<0.05), suggesting that increased CSH levels stimulate muscle hypertrophy, potentially due to the growth hormone-enhancing effects of CSH [7]. These findings align with previous studies, which have shown that CSH supplementation enhances fiber density and modifies fiber type distribution [29].

### Fatty acid profile

The fatty acid profiles of beef from steers fed diets supplemented with different levels of CSH are presented in Table 7. The composition of fatty acids is critical in determining meat quality, influencing the texture of adipose tissue, oxidative stability of muscle, and ultimately flavor and color [36]. CSH supplementation resulted in significant changes to the fatty acid composition across the dietary treatments. However, no significant differences (p>0.05) were observed in the levels of decanoic acid (C10:0), myristoleic acid (C14:1), palmitoleic acid (C16:1), heptadecenoic acid (C17:1), and octadecanoic acid (C18:0).

The proportions of dodecanoic acid (C12:0), tetradecanoic acid (C14:0), pentadecanoic acid (C15:0), hexadecanoic acid (C16:0), linoleic acid (C18:2), linolenic acid (C18:3), eicosanoic acid (C20:0), eicosadienoic acid (C20:2), eicosatrienoic acid (C20:3), and arachidonic acid (C20:4) were significantly lower in the 1.0% CSH-supplemented group (p<0.05). Additionally, these fatty acids, along with conjugated linolenic acid (C18:2c9t11), showed a linear decrease with increasing CSH levels in the diet (p<0.05). These reductions likely reflect a suppression of de novo fatty acid synthesis, potentially driven by CSH’s influence on growth hormone regulation and metabolic pathways [37]. As growth hormone and other pituitary factors play a critical role in lipid mobilization [29], CSH may influence the fatty acid profile of the LD muscle by stimulating growth hormone secretion. Conversely, oleic acid (C18:1), eicosenoic acid (C20:1), and monounsaturated fatty acids (MUFA) were significantly higher in the 1.0% CSH-supplemented group (p<0.05), and also exhibited a linear increase with increasing CSH concentrations (p<0.05). In contrast, the proportions of polyunsaturated fatty acids (PUFA) and saturated fatty acids were significantly lower in the 1.0% CSH-supplemented group (p<0.05), showing a linear decrease with higher CSH concentrations (p<0.05). These changes are likely attributable to CSH’s effect on ruminal biohydrogenation, which may modulate microbial activity and facilitate the conversion of PUFA to either saturated or MUFAs [6]. Such modifications in fatty acid composition could enhance the nutritional profile and commercial appeal of beef products.

## CONCLUSION

This study concludes that dietary supplementation with 1.0% CSH effectively enhances growth rate, carcass traits, and meat quality in steers. Significant improvements were observed in BWG, ADG during the 150-day feeding period. Additionally, DP, carcass weight, and shear force value showed positive changes, along with favorable modifications in muscle fiber composition and fatty acid profiles. The inclusion of 1.0% CSH in the concentrate was identified as the optimal feeding dose. These findings emphasize the potential of CSH as an effective dietary strategy to improve both production efficiency and meat quality in beef cattle.

## Figures and Tables

**Table 1:** Feed ingredients and chemical compositions of experimental diets (% DM)

Item	Control	0.5% CSH	1.0% CSH
Cassava chip	57.6	57.1	57.1
Rice bran	6.2	6.2	5.7
Lucerne meal	3.0	3.0	3.0
Palm kernel meal	15.0	15.0	15.0
Soybean meal	5.0	5.0	5.0
Molasses	8.0	8.0	8.0
Urea	1.8	1.8	1.8
Trace mineral	0.5	0.5	0.5
Salt	1.0	1.0	1.0
Dicalcium phosphate	1.8	1.8	1.8
Sulfur	0.1	0.1	0.1
Coated cysteamine HCl	0.0	0.5	1.0
Total	100.0	100.0	100.0
Chemical compositions (%DM)			
DM	88.4	88.4	88.5
CP	12.0	12.0	12.0
EE	1.6	1.6	1.5
NDF	12.5	12.5	12.2
ADF	9.7	9.7	9.5
Ash	7.6	7.5	7.8
TDN	72.5	72.1	71.7

DM, dry matter; CSH, coated cysteamine hydrochloride; CP, crude protein; EE, ether extract; NDF, neutral detergent fiber; ADF, acid detergent fiber; TDN, total digestible nutrients.

**Table 2:** Least squares means for dry matter intake and growth performance of steers fed a diet supplemented with different levels of coated cysteamine HCl

Item	Coated cysteamine HCl (%DM)			SEM	p-value^1)^		
	
	0.0	0.5	1.0		T	L	Q
DMI (kg/day)	11.9	12.3	12.7	0.20	0.40	0.18	0.99
DMI/BW (%)	2.4	2.4	2.4	0.04	0.83	0.58	0.81
Feed conversion ratio	13.6	12.2	10.7	0.45	0.13	0.04	0.99
Total feed cost (USD/day)	2.8^c^	3.2^b^	3.6^a^	0.04	<0.01	<0.01	0.68
Cost/gain (USD/kg)	3.2	3.2	3.0	0.10	0.83	0.61	0.76
Live weight (kg)							
30 d	455.8	453.7	459.8	7.22	0.78	0.68	0.59
60 d	482.5^b^	488.7^b^	516.2^a^	8.31	0.02	0.01	0.27
90 d	515.5^y^	532.7^xy^	552.2^x^	8.81	0.06	0.02	0.92
120 d	538.5^b^	561.5^b^	595.7^a^	9.78	<0.01	<0.01	0.62
150 d	576.7^b^	589.1^b^	629.2^a^	9.68	0.01	<0.01	0.26
200 d	600.7^y^	625.7^y^	657.8^x^	11.23	0.05	0.02	0.82
Average daily gain (kg/d)							
0–30 d	1.26	1.19	1.39	0.12	0.78	0.69	0.59
0–60 d	1.07^b^	1.18^b^	1.64^a^	0.08	0.02	0.01	0.27
0–90 d	1.08^y^	1.27^xy^	1.49^x^	0.06	0.06	0.02	0.91
0–120 d	1.01^b^	1.20^b^	1.48^a^	0.04	<0.01	<0.01	0.61
0–150 d	1.06^b^	1.14^b^	1.41^a^	0.04	0.01	<0.01	0.26
0–200 d	0.93	1.05	1.18	0.04	0.11	0.04	0.89

1) T, the treatment effect of dietary CSH addition in concentrate; L, the linear effects of increasing CSH levels in concentrate; Q, the quadratic effects of increasing CSH levels in concentrate.

a–c Means within the same row with different superscripts differ significantly (p<0.05).

x,y Means within the same row with different superscripts indicate a trend towards a difference (p<0.^1)^.

DM, dry matter; SEM, standard error of the means; DMI, dry matter intake; DMI/BW; dry matter intake as percent of body weight; Cost/gain, feed cost per kilogram of gain (USD/kg); CSH, coated cysteamine hydrochloride.

**Table 3:** Least squares means for carcass characteristics of steers fed a diet supplemented with different levels of coated cysteamine HCl

Item	Coated cysteamine HCl (%DM)			SEM	p-value^1)^		
	
	0.0	0.5	1.0		T	L	Q
Hot carcass weight (kg)	327.6^b^	333.6^b^	374.2^a^	4.44	0.01	<0.01	0.09
Cold carcass weight (kg)	315.8^b^	323.3^b^	362.0^a^	4.29	0.01	<0.01	0.12
Dressing percentage (%)	54.7^ab^	53.2^b^	56.5^a^	0.47	0.04	0.18	0.03
Rib-Eye area (cm^2^)	88.0	95.6	94.1	3.39	0.60	0.47	0.47
Back fat thickness (cm)	0.9	0.6	1.0	0.13	0.46	0.77	0.24
Marbling^2)^	2.2	2.1	2.2	0.06	0.78	0.76	0.54
Sheer force (N)	97.9^a^	94.2^a^	50.7^b^	7.10	0.03	0.02	0.21
Cooking loss (%)	35.9^a^	33.5^ab^	31.1^b^	0.46	<0.01	<0.01	0.98
Chilling loss (%)	3.7	3.1	3.3	0.18	0.40	0.50	0.24
Longissimus color^3)^							
L*	43.4^b^	43.7^b^	50.2^a^	0.81	<0.01	<0.01	0.09
a*	13. 8^x^	14.0^x^	11.8^y^	0.36	0.06	0.81	0.02
b*	12.0	13.1	13.2	0.25	0.14	0.07	0.39

1) T, the treatment effect of dietary CSH addition in concentrate; L, the linear effects of increasing CSH levels in concentrate; Q, the quadratic effects of increasing CSH levels in concentrate.

2) 1, devoid; 2, trace; 3, slight; 4, moderate; 5, abundant.

3) L*, lightness; a*, redness; b*, yellowness.

a,b Means within the same row with different superscripts differ significantly (p<0.05).

x,y Means within the same row with different superscripts indicate a trend towards a difference (p<0.^1)^. DM, dry matter; SEM, standard error of the means; CSH, coated cysteamine hydrochloride.

**Table 4:** Least squares means for chemical composition and muscle fiber characteristics of steers fed a diet supplemented with different levels of coated cysteamine HCl

Item	Coated cysteamine HCl (%DM)			SEM	p-value^1)^		
	
	0.0	0.5	1.0		T	L	Q
Chemical composition (%)							
Moisture	71.2	70.4	69.5	0.32	0.11	0.04	0.88
Crude protein	23.3	24.1	23.2	0.16	0.11	0.91	0.04
Fat	7.1	7.5	7.4	0.10	0.14	0.12	0.19
Ash	1.0	1.1	1.1	0.01	0.26	0.35	0.18
Muscle fiber characteristics (μm)							
Sarcomere length	1.56	1.7	1.6	0.03	0.10	0.81	0.04
Muscle fiber diameter	38.4^b^	37.7^b^	48.5^a^	1.72	0.02	0.02	0.10

1) T, the treatment effect of dietary CSH addition in concentrate; L, the linear effects of increasing CSH levels in concentrate; Q, the quadratic effects of increasing CSH levels in concentrate.

a,b Means within the same row with different superscripts differ significantly (p<0.05).

DM, dry matter; SEM, standard error of the means; CSH, coated cysteamine hydrochloride.

**Table 5:** Least squares means for subprimal cut of steers fed a diet supplemented with different levels of coated cysteamine HCl

Item (% of chilled carcass)	Coated cysteamine HCl (%DM)			SEM	p-value^1)^		
	
	0.0	0.5	1.0		T	L	Q
Rib set	5.3^b^	6.3^a^	6.4^a^	0.10	<0.01	<0.01	0.05
Chuck	3.9	4.3	3.7	0.17	0.37	0.53	0.20
Macreuse (clod)	3.4^a^	3.2^ab^	2.9^b^	0.07	0.03	<0.01	0.60
Paleron (flat iron)	1.7^a^	1.4^b^	1.2^b^	0.04	<0.01	<0.01	0.48
Fore shank	1.7^a^	1.4^b^	1.2^b^	0.03	<0.01	<0.01	0.08
Brisket	4.7	4.6	4.8	0.11	0.77	0.87	0.48
Short rib	2.7	2.9	2.8	0.04	0.19	0.52	0.10
Silver shank	0.4^a^	0.4^b^	0.3^b^	0.01	<0.01	<0.01	0.24
Stew meat	12.3^b^	14.5^a^	15.8^a^	0.38	0.01	<0.01	0.61
Hampe	0.3	0.3	0.3	0.01	0.47	0.29	0.53
T-bone	7.5^b^	7.5^b^	8.4^a^	0.09	<0.01	<0.01	0.02
Sirloin	3.9	4.0	4.1	0.09	0.74	0.46	0.89
Tritip	0.8	0.8	0.8	0.02	0.36	0.19	0.66
Top round	6.0	5.8	5.5	0.12	0.19	0.08	0.71
Knuckle	4.3^a^	3.9^ab^	3.3^b^	0.13	0.02	<0.01	0.67
Bottom round	5.6^a^	5.0^b^	5.0^b^	0.11	0.04	0.03	0.20
Hide shank	1.6^a^	1.3^b^	1.2^b^	0.05	0.01	<0.01	0.39
Nerveux (blade)	1.8^a^	1.5^b^	1.3^b^	0.04	<0.01	<0.01	0.45
Bavette aloyau (flap)	0.8	0.8	0.8	0.02	0.27	0.13	0.56
Bavette flanchet (flank)	0.4	0.4	0.4	0.02	0.99	0.90	0.91
Bone	15.3	15.6	15.8	0.84	0.97	0.83	0.95
Scraps	3.3^a^	1. 6^b^	1.2^c^	0.05	<0.01	<0.01	<0.01
Tendon	1.3^a^	0.9^b^	1.0^b^	0.06	0.05	0.03	0.10
Fat	10.7	11.5	12.3	0.28	0.18	0.07	0.91

1) T, the treatment effect of dietary CSH addition in concentrate; L, the linear effects of increasing CSH levels in concentrate; Q, the quadratic effects of increasing CSH levels in concentrate.

a,b Means within the same row with different superscripts differ significantly (p<0.05).

DM, dry matter; SEM, standard error of the means; CSH, coated cysteamine hydrochloride.

**Table 6:** Least squares means for non-carcass weight of steers fed a diet supplemented with different levels of coated cysteamine HCl

Item (kg)	Coated cysteamine HCl (%DM)			SEM	p-value^1)^		
	
	0.0	0.5	1.0		T	L	Q
Feet	10.7^b^	12.7^ab^	13.3^a^	0.34	0.03	0.01	0.31
Head	21.7^b^	23.3^ab^	24.8^a^	0.29	0.01	<0.01	0.96
Skin	38.0	41.5	40.8	1.34	0.60	0.47	0.47
Digestive tract	17.5^b^	21.6^a^	20.6^ab^	0.48	0.02	0.04	0.03
Liver	4.4^y^	5.2^x^	5.0^xy^	0.10	0.06	0.08	0.09
Tail	1.8	1.9	1.7	0.06	0.56	0.52	0.40
Scraps	1.5^b^	1.8^a^	1.8^a^	0.04	0.02	0.03	0.03
Bile	0.3	0.3	0.3	0.03	0.72	0.78	0.47

1) T, the treatment effect of dietary CSH addition in concentrate; L, the linear effects of increasing CSH levels in concentrate; Q, the quadratic effects of increasing CSH levels in concentrate.

a,b Means within the same row with different superscripts differ significantly (p<0.05).

x,y Means within the same row with different superscripts indicate a trend towards a difference (p<0.^1)^.

DM, dry matter; SEM, standard error of the means; CSH, coated cysteamine hydrochloride.

**Table 7:** Least squares means for fatty acid profile of steers fed a diet supplemented with different levels of coated cysteamine HCl (% of total fatty acid)

Item	Coated cysteamine HCl (%DM)			SEM	p-value^1)^		
	
	0.0	0.5	1.0		T	L	Q
Decanoic acid (C10:0)	0.04	0.04	0.03	<0.01	0.16	0.20	0.14
Dodecanoic acid (C12:0)	0.3^a^	0.1^b^	0.1^b^	0.02	<0.01	<0.01	0.04
Tetradecanoic acid (C14:0)	4.6^a^	4.1^ab^	3.5^b^	0.14	0.03	<0.01	0.75
Myristoleic acid (C14:^1)^	0.9	1.1	1.0	0.07	0.79	0.70	0.59
Pentadecanoic acid (C15:0)	0.5^a^	0.3^b^	0.3^b^	0.02	<0.01	<0.01	0.23
Hexadecanoic acid (C16:0)	30.2^a^	30.7^a^	28.2^b^	0.37	0.04	0.05	0.09
Palmitoleic acid (C16:^1)^	4.3	4.3	4.2	0.16	0.92	0.69	0.96
Heptadecenoic acid (C17:^1)^	0.7	0.7	0.7	0.04	0.76	0.64	0.58
Octadecanoic acid (C18:0)	14.7	14.8	13.7	0.40	0.46	0.31	0.49
Oleic acid (C18:^1)^	37.6^c^	41.6^b^	46.2^a^	0.51	<0.01	<0.01	0.82
Linoleic acid (C18:2)	3.2^a^	1.2^b^	1.1^b^	0.25	<0.01	<0.01	0.09
Linolenic acid (C18:3)	0.2^a^	0.1^b^	0.1^b^	0.01	<0.01	<0.01	0.06
CLA (C18:2c9t1^1)^	0.3^x^	0.3^xy^	0.2^y^	0.02	0.07	0.03	0.73
Eicosanoic acid (C20:0)	0.13^a^	0.11^ab^	0.08^b^	0.01	0.01	<0.01	0.81
Eicosenoic acid (C20:^1)^	0.2^b^	0.2^b^	0.3^a^	0.01	<0.01	<0.01	<0.01
Eicosadienoic acid (C20:2)	0.2^a^	0.1^b^	0.1^b^	0.03	0.04	0.03	0.21
Eicosatrienoic acid (C20:3)	0.4^a^	0.2^b^	0.1^b^	0.04	0.04	0.02	0.35
Arachidonic acid (C20:4)	1.5^a^	0.3^b^	0.2^b^	0.19	0.02	0.01	0.17
MUFA	43.7^c^	47.7^b^	52.4^a^	0.55	<0.01	<0.01	0.81
PUFA	5.9^a^	2.1^b^	1.8^b^	0.49	0.01	<0.01	0.12
SFA	50.4^a^	50.2^b^	45.9^b^	0.55	<0.01	<0.01	0.11

1) T, the treatment effect of dietary CSH addition in concentrate; L, the linear effects of increasing CSH levels in concentrate; Q, the quadratic effects of increasing CSH levels in concentrate.

a–c Means within the same row with different superscripts differ significantly (p<0.05).

DM, dry matter; SEM, standard error of the means; CLA, conjugated linolenic acid; MUFA, monounsaturated fatty acids; PUFA, polyunsaturated fatty acids; SFA, saturated fatty acids; CSH, coated cysteamine hydrochloride.
